# Allogenic Adipose Derived Stem Cells Transplantation Improved Sciatic Nerve Regeneration in Rats: Autologous Nerve Graft Model

**DOI:** 10.3389/fphar.2018.00086

**Published:** 2018-03-06

**Authors:** Ruslan Masgutov, Galina Masgutova, Liliya Mukhametova, Ekaterina Garanina, Svetlana S. Arkhipova, Elena Zakirova, Yana O. Mukhamedshina, Zhuravleva Margarita, Zarema Gilazieva, Valeriia Syromiatnikova, Adelya Mullakhmetova, Gulnaz Kadyrova, Mariya Nigmetzyanova, Sergeev Mikhail, Pankov Igor, Ramil Yagudin, Albert Rizvanov

**Affiliations:** ^1^OpenLab “Gene and Cell Technologies”, Institute of Fundamental Medicine and Biology, Kazan Federal University, Kazan, Russia; ^2^Republican Clinical Hospital, Kazan, Russia; ^3^Department of Histology, Cytology and Embryology, Kazan State Medical University, Kazan, Russia; ^4^Kazan State Academy of Veterinary Medicine, Kazan, Russia; ^5^Department of Traumatology and Orthopedics, Kazan State Medical Academy, Kazan, Russia

**Keywords:** PNI, autologous nerve graft, DRG, PCR, myelin fibers, IVIS Spectrum

## Abstract

We examined the effect of transplantation of allogenic adipose-derived stem cells (ADSCs) with properties of mesenchymal stem cells (MSCs) on posttraumatic sciatic nerve regeneration in rats. We suggested an approach to rat sciatic nerve reconstruction using the nerve from the other leg as a graft. The comparison was that of a critical 10 mm nerve defect repaired by means of autologous nerve grafting versus an identical lesion on the contralateral side. In this experimental model, the same animal acts simultaneously as a test model, and control. Regeneration of the left nerve was enhanced by the use of ADSCs, whereas the right nerve healed under natural conditions. Thus the effects of individual differences were excluded and a result closer to clinical practice obtained. We observed significant destructive changes in the sciatic nerve tissue after surgery which resulted in the formation of combined contractures in knee and ankle joints of both limbs and neurotrophic ulcers only on the right limb. The stimulation of regeneration by ADSCs increased the survival of spinal L5 ganglia neurons by 26.4%, improved sciatic nerve vascularization by 35.68% and increased the number of myelin fibers in the distal nerve by 41.87%. Moreover, we have demonstrated that S100, PMP2, and PMP22 gene expression levels are suppressed in response to trauma as compared to intact animals. We have shown that ADSC-based therapy contributes to significant improvement in the regeneration.

## Introduction

The major pathogenesis element in the case of peripheral nerve injury is the destruction of neurons. Neurons form complex connections to transfer information from peripheral receptors of sensory neurons to an organ and back to the CNS that provides sensory and motor functions. The peripheral nerve that acts as a conductor consists of a neuronal axon, Schwann cells, fibroblasts as well as elements of nerve blood supply. The key cell type for peripheral nerve regeneration is the Schwann cells which form axial cylinders that support and guide axons during post-injury nerve regeneration.

A peripheral myelin protein-22 (PMP22) which is involved in the formation of a myelin sheath is one of the key proteins involved in its post-traumatic repair. The molecular architecture of myelinated nerve fibers is of importance for the functional integrity of peripheral nerves and is often impaired in peripheral nerve disorders (Li et al., 2013). PMP22, as an integral membrane glycoprotein of intermodal myelin, is estimated to contain 2–5% of the total number of myelin proteins in the peripheral nervous system (Snipes et al., 1992) and is normally expressed in the Schwann cells (Li et al., 2013). This expression is dose-dependent, and impairments are related to hereditary demyelinating neuropathies. With a genetic low expression myelination of peripheral nerves is delayed (Amici et al., 2006). The interactions with an extracellular domain of the protein zero (P0, Mpz) are one of the mechanisms through which PMP22 might affect myelin stability (Li et al., 2013). The protein P2 (PMP2) is another important protein directly involved in regeneration processes. PMP2 is a myelin protein binding fatty acids (FABP) which is expressed in large amounts in peripheral nerve myelin sheaths (Furuhashi and Hotamisligil, 2008). It is also known that the protein binds to the lipid surface of the membrane causing its folding (Suresh et al., 2010). It also influences changes in double-layer myelin lipid membranes (Knoll et al., 2010). In the sciatic nerve as well as in dorsal and ventral radices of the spinal ganglia of an adult animal, unequal distribution of P2 protein including its absence in some myelin axons can be seen (Zenker et al., 2014). Similar studies with gene PMP2 knockout mice revealed no changes in the overall structure of the myelin sheath but this change had an impact on the level of lipids in the myelin sheath. Studies with the mutated gene PMP2 in demyelinating neuropathies show interdependence between PMP2 and PMP22 genes (Hong et al., 2016).

The issues of peripheral nerve regeneration drew the attention of researchers to S100 gene mRNA expression values in different types of injury. The members of the S100 protein family are small dimer calcium-binding proteins. They contain only amino acid sequences which are highly conserved in vertebrates (Quincozes-Santos and Gottfried, 2011; D’Angelo et al., 2012). S100 proteins can form both homo- and heterodimers; in addition to Ca2+ they can also bind Zn2+ and Cu2+. Ion capture changes a spatial organization (Liu et al., 2013). A nerve injury induces a system of regeneration *in vivo*, including the excessive expression of the S100 protein and initiating neuroregeneration (Voronina et al., 2009; Liu et al., 2013).

Regeneration after nerve injury is a complex process associated with inflammation, adhesion, regulation of neurotrophic factors, neurotransmitter synthesis and release, the formation of a growth cone and axonal growth as well as neuron survival (Angius et al., 2012; Chen et al., 2012; Ozdemir et al., 2012; Oh et al., 2013). At the same time, excessive immune responses can cause the expression of inflammatory mediators around a damaged tissue. They promote the formation of neuromas and hyperplasia which directly affect the restoration of conduction after nerve injury (Schachtrup et al., 2010). An excessive immune inflammatory response in turn inhibits S100 protein expression (Michetti and Gazzolo, 2002). In the case of peripheral nerve transection, along with axon injury, regeneration is complicated by ischemia and hypoxia due to vascular injury at the trauma site, and complete regeneration is impossible without the restoration of these vessels (Trehan et al., 2016). In spite of regeneration of sensory neuron axons, major peripheral nerve traumas may cause paralysis of the entire extremity or its distal parts (Massing et al., 2010; Ronchi and Raimondo, 2017). Therefore, complex reconstruction providing vascularization of the injured area, survival of sensory neurons of the spinal ganglion, restoration of the structure and function of the injured nerve including axon growth and Schwann cell proliferation necessary for remyelination as well as restoration of nerve fiber conduction are needed to achieve adequate limb function after a peripheral nerve injury (Sullivan et al., 2016).

The use of microsurgical methods and modern suture material minimizes the risk of additional injury during interventions on nervous trunks; nevertheless, the restoration of the limb function often fails because the autologous reserves are limited. To solve this problem, injured nerve regeneration is stimulated using growth and trophic factors as well as stem cells from various sources (de Luca et al., 2015) that show neuroprotective effect; another option is gene therapy (Hoyng et al., 2015).

Recent studies dedicated to tissue regeneration showed the effectiveness and safety of the use of cells that allow relatively simple *in vitro* production of variously differentiated populations to facilitate and accelerate tissue regeneration (de Luca et al., 2015). According to the literature, the use of adipose tissue-derived stem cells may be promising, as sufficient quantities of stem cells can be derived in a relatively short time (Salehi et al., 2016).

Adipose-derived stem cell (ADSCs) have similar properties to MSCs according to the expression of mesenchymal stem cell (MSC) basic markers (Akbulut et al., 2012). MSCs have a ability of *trans*-differentiation (Odabaş et al., 2008; Wakao et al., 2012), and ADSCs exhibited the same differentiation capacity compared to bone marrow stem cells, bone, cartilage, adipocytes and skeletal muscle that originated from mesoderm. This is why ADSCs began to be widely used by clinicians in tissue engineering (Dai et al., 2016).

It has been shown that ADSCs facilitate functional and structural recovery of an injured peripheral nerve (Erba et al., 2010; Masgutov et al., 2016). The ability of ADSCs to express large quantities of transcripts that code proteins participating in different stages of neurogenesis and neuroregeneration has also been demonstrated. Due to the expression of such proteins, ADSCs may have an impact on different stages of structural and functional recovery of an injured nerve, from axon growth and Schwann cell proliferation to nerve fiber myelination and restoration of their conductivity (Heine et al., 2004; Carlson et al., 2011; Zack-Williams et al., 2015).

Many different animal and injury models have been used to test different approaches to peripheral nerve injury (Dalamagkas et al., 2016). However, generally accepted and widely used experimental models of peripheral nerve traumatic injury where the defect is repaired with an autologous nerve are, in our opinion, a far cry from clinical practice. The common experimental model of autologous nerve grafting is to transect the nerve with two transverse sections with subsequent approximation of the transected fragments using an autologous freshly dissected nerve, the ends being sutured with epineural sutures. In such a case fascicles are approximated which makes it easier for regenerating axons to grow through already formed pathways. The model we propose is aimed at replacement of a portion of the nerve by a fragment of nervous tissue on the right side of the sciatic nerve which is closer to clinical experience because of an anatomic feature of mammals involving a difference between contralateral sides.

Further, in the model we propose the same animal acts both as a test model and control where the left side nerve regeneration is facilitated by the use of ADSCs, and on the right side the healing process is left unaided. Thus we exclude individual differences of the animals and obtain results that are closer to clinical experience.

## Materials and Methods

All procedures in animals were approved by the Kazan Federal University Animal Care and Use Committee (Permit Number: 5 dated 27 May 2014) and carried out in accordance with international bioethical standards.

### Experiments *in Vitro*

Adipose-derived stem cells were obtained using the standard method from the adipose tissue of healthy Wistar rats by incubation with crab collagenase (Biolot) (Zakirova et al., 2015). The immunophenotype of the obtained cells was assessed based on the expression of a number of surface markers: CD 29 (BioLegend, No. 102208, 1:100), CD 44 (BioLegend, No. 103028, 1:100), Stro-1 (BioLegend, No. 340104, 5:100), Thy-1 (BioLegend, No. 328112, 1:100), CD 34 (Santa Cruz, sc-7324, 1:100). Prior to visualization, the sections were incubated with corresponding fluorophore-conjugated secondary antibodies for 2 h at RT. Cells were stained according to the protocols of manufacturing companies. For the staining of nuclei the dye DAPI (Sigma, United States) at the concentration of 1 μg/ml was used. The slides were analyzed using a Carl Zeiss confocal microscope (Germany). After immunophenotype determination the cells were transduced with lentivirus coding the eGFP gene (LV-eGFP). For the transplantation only ADSCs expressing EGFP were used, obtained by sorting on a flow cytofluorometer FACS Aria III (BD).

### Animal Experiments

Animal experiments were carried out in 26 white male Wistar rats (Pushchino Laboratory, Russia). Animals weighed 300–400 g and were 5–6 months old. Prior to the experiment and for 60 days after surgery, the animals were housed under standard conditions with food and water *ad libitum*. Nerves in 6 out of 26 experimental animals were left intact. 20 randomly selected animals underwent autoplasty of the sciatic nerve.

**FIGURE 1:**
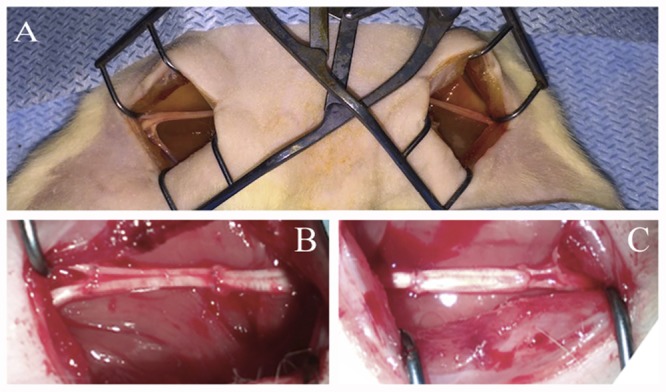
Autologous rat sciatic nerve graft. **(A)** Open wounds to sciatic nerves. **(B)** Autologous left 10 mm, sciatic nerve grafting using the right sciatic nerve fragment stimulated by ADSCs. **(C)** Autologous right 10 mm, sciatic nerve grafting using the left sciatic nerve fragment without additional cells.

Under aseptic conditions and anesthesia with 6.4% chloral hydrate solution (400 mg/kg) the sciatic nerves were transected at midthigh level. Nerves were transected with two cross sections forming a critical 10 mm defect which constitutes 25% of the entire length of a rat sciatic nerve in an average animal. The ends of the nerve were approximated using an autologous nerve graft — a portion of the contralateral nerve – taking into account the nerve growth cone and then suturing it with Prolene 10.0 using 4 interrupted epineural sutures (**Figure 1**).

After suturing the nerve portions through a graft both nerves were covered with the fibrin glue Tissucol-Kit (Baxter AG, Austria) (Tissucol). In the left nerve Tissucol contained 1 × 10^6^ of ADSCs-LV-eGFP. The right sciatic nerve was covered with Tissucol alone. The wound was closed in layers using Prolene 4.0 sutures. Antibiotics and analgesics were administered as follows: 1 mL gentamicin (25 mg/kg, Omela, Russian Federation) was injected intramuscularly for 7 consecutive days; buprenorphine (0.5 mg/kg) was injected subcutaneously for about 7 days after surgery to minimize pain.

### Evaluation of Cell Migration

Fourteen days after injury the migration of transplanted eGFP-expressing ADSCs was evaluated with a non-invasive method using the IVIS Spectrum system (PerkinElmer, Inc.). A 3D tomographic reconstruction was built with a device which determined the location of ADSCs and evaluated their migration. Prior to the evaluation the fur from the lower body of rats was removed with a depilatory cream in order to reduce autofluorescence. Visualization was carried out under the manufacturer’s protocol.

### Evaluation of Nerve Vascularization

Sixty days after surgery the evaluation of blood flow restoration in the area of injury to the left and right sciatic nerves was carried out by means of real time microcirculation visualization using the laser Doppler EasyLDI (Aimago, Switzerland) in anesthetized animals. The laser beam of the device was pointed to the distal portion of the nerve, using blood flow estimation the changes of the microcirculation values were analyzed in real time. The measurement was done in absolute perfusion units (Apu) under the manufacturer’s protocol.

### Material Collection and Evaluation of S100, PMP22, and PMP2 mRNA Expression

Thirty and sixty days after the surgery the L4–L6 spinal ganglia as well as sciatic nerves on the left and right operated sides were sampled. To isolate spinal ganglia, laminectomy was carried out at the L4–L6 level, and an operative approach to the relevant DRG of the left and right sides provided. To isolate the sciatic nerve access was gained at the midthigh level within an area of its reconstruction. These tissues were used to quantitatively analyze the expression of S100, PMP2, PMP22 genes by means of real-time PCR (polymerase chain reaction) using the CFX 96 Real Time PCR Detection system (Bio-Rad, United States). Isolation of total RNA from the portions of rat sciatic nerves and spinal ganglia was carried out using the kit by Yellow Solve (Silex) according to the manufacturer’s protocol. For the synthesis of the DNA a complementary strand 1 μg of RNA template, 20 U of RNase inhibitor, 200 U of reverse transcriptase and 100 pmol of a random hexaprimer were used. Temperature conditions of reverse transcription were as follows: preheating -25°C, synthesis -42°C, transcription termination was carried out at 70°C for 10 min. Real time PCR was conducted using oligonucleotide primers and fluorescent probes (Taqman^®^).

For real time PCR in a volume of 10 μl the following components were used: 2.5X reaction mixture {[KCl, Tris-HCl (pH 8.8), 6.25 mM of MgCl_2_], Taq DNA-polymerase, deoxynucleotide triphosphates, glycerol, Tween 20}, 900 nM of forward and reverse primers, 300 nM of the probe labeled with fluorochrome (carboxyfluorescein, FAM) at 5′-end and bearing the quencher RTQ-1 on its 3′-end (from England “real-time quenchers”, 520 nm), water (Synthol) and 1 μl of template DNA (cDNA). Primer kit and a probe for the analysis of the expression level of gene 18 s of rRNA were used as controls. Primer and sample sequences are shown in **Table 1**. Ready reaction mixture 10 μl in 96-well plates (Axygen, 96 Well Full Skirt PCR Microplates), 0.5 μl of cDNA was added to each well, the wells covered with an optically transparent film (Axygen, Sealing Film, Real Time PCR), and then PCR amplification was carried out according to the TaqMan 2Step.tmo protocol: 95°C for 30 s (calibration), 95°C for 3 min (polymerase activation and denaturation of ligated antibodies), then 45 cycles at 95°C for 15 s and at 55°C for 30 s.

**Table 1:** Nucleotide sequences of primers and samples for real time PCR.

Name	Sequence 53
rmhS100 — F	ATgTCTTCCATCAgTATTCAggg
rmhS100 — R	TCTCCATCACTTTgTCCACC
rmhS100 — pr	[FAM] TCTTCAgCTTgTgCTTgTCACCCT [BH1]
Pmp22 — TM — S	gTgCTAgTgTTgCTCTTC
Pmp22 — TM — A	ggATgTggTACAgTTCTg
Pmp22 — TMpr	[FAM] CTCCACCATCgTCAgCCAAT [BH1]
rPmp2 — TM — S	ggAgACTATATCACCATTAgA
rPmp2 — TM — A	TCCAgCAACTTTCTCTTTA
Pmp2 — TMpr	[FAM] CCACTTCTgCACTTgCTTCA [BH1]
rmh18S — TMF	gCCgCTAgAggTgAAATTCTTg
rmh18S — TMR	CATTCTTggCAAATgCTTTCg
rmh18S TMpr	[HEX] ACCggCgCAAgACggACCAg [BH2]

The quantity of RNA was normalized against the quantity of cDNA of the gene 18s rRNA. The serial dilution of cDNA synthesized from mRNA of the sciatic nerve and spinal ganglia of intact animals was used for plotting a standard curve and determining the gene expression level.

### Morphology and Morphometry

Sixty days after the autologous grafting the number of sensory neurons that survived after the injury was counted in the spinal ganglia L5 on both experimental and control sides. Distal portions of the sciatic nerve were used to determine the number of regenerating myelin fibers.

To determine the quantity of surviving neurons the isolated L5 spinal ganglia were fixed in 40% neutral formalin and embedded in paraffin using the standard technique. 7 μm thick sections were prepared from the specimens with a Automatic microtome HM 355S (Thermo Fisher Scientific). Total number of surviving neurons was assessed on stained slides with azure-eosin (5%, Minimed, Russia). Neurons with nucleoli were visualized under a Carl Zeiss Primo Star Microscope (Carl Zeiss) with the 40-fold magnification.

For transmission electron microscopy in 60 days after the injury a sciatic nerve section was taken 5–7 mm from the distal suture line and fixed in 2,5% glutaraldehyde in 1 M phosphate buffer, post fixed in 1% osmium tetroxide, dehydrated in ethanol from 30 to 96%, acetone and then propylene oxide, and finally embedded in Epon 812 resin. After polymerization at 37, 45, and 60°C, samples were cut into semithin (1 mkm) and ultrathin (∼0,1 mkm) sections using ultramicrotome (Leica UC7, Germany). Sections were mounted on Cu grids (200 mesh, Sigma) and contrasted with 1% uranyl acetate (10 min at 60°C) and lead citrate (10 min at RT). Ultrathin sections for morphological analysis were examined using Transmission Electron Microscope HT7700 (Hitachi, Japan) at 100 kV.

Semifine sections were used to count the number of myelin fibers. A random sampling of four nerve portions were analyzed under a Primo Star Microscope (Carl Zeiss) with the 63 ×100 magnification and immersion in oil.

### Statistical Analysis

Data are presented as mean ± standard error mean (SEM). A one-way analysis of variance (ANOVA) with Tukey’s test was used for multiple comparisons between all experimental groups. All analyses were performed in a blinded manner with respect to the treatment group. A value of *P* < 0.05 was considered statistically significant. Data were analyzed using the Origin 7.0 SR0 Software (OriginLab, Northampton, MA, United States).

## Results

**FIGURE 2:**
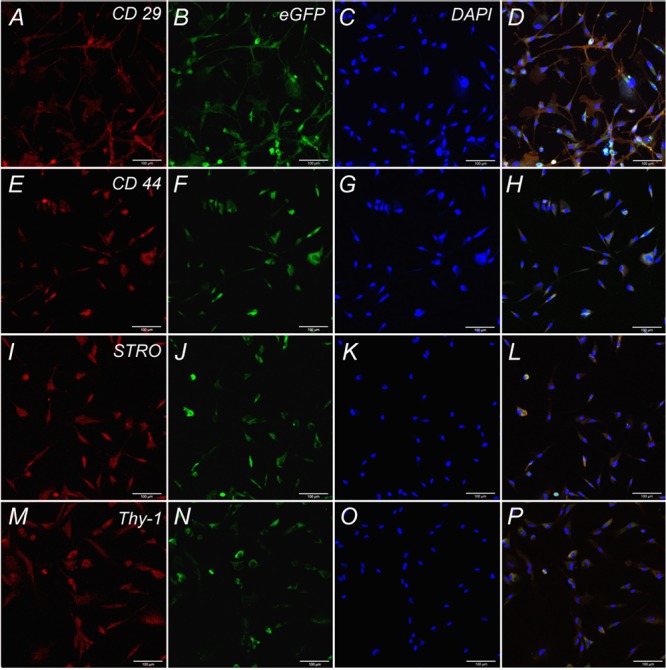
Expression of CD 29, CD 44, STRO, Thy-1 by undifferentiated ADSCs subpopulations. Row 1: Confocal analysis of the expression of CD 29 **(A)**, e-GFP **(B)**, DAPI **(C)**, Merge **(D)**. Row 2: Confocal analysis of the expression of CD 44 **(E)**, e-GFP **(F)**, DAPI **(G)**, Merge **(H)**. Row 3: Confocal analysis of the expression of STRO **(I)**, e-GFP **(J)**, DAPI **(K)**, Merge **(L)**. Row 4: Confocal analysis of the expression of Thy-1 **(M)**, e-GFP **(N)**, DAPI **(O)**, Merge **(P)**. Bar: 100 μm.

Cells isolated from rat adipose tissue had a fibroblast-like morphology and high proliferative activity. They expressed membrane markers typical for MSCs (CD 29, CD 44, Stro-1, Thy-1) and did not express the hematopoietic stem cell marker CD 34 (**Figure 2**).

**FIGURE 3:**
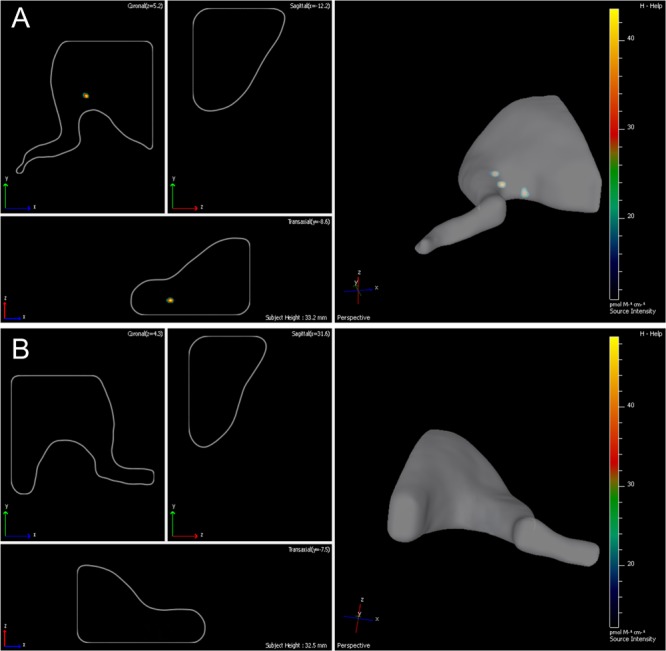
Lifetime assessment survival and migration of eGFP-ADSCs using the IVIS Spectrum system. Green fluorescence indicates the ADSCs survival and migration on the experimental side (left side) **(A)**. There is no fluorescence on the contralateral control side (right side) **(B)** 14 days after the operation.

Fourteen days following the surgery -autologous nerve graft and transplantation of ADSCs expressing green fluorescent protein- the inserted cells were localized with the help of the IVIS Spectrum system. The right limb served as control. We showed that grafted cells are found predominantly in the transplantation area, their partial retrograde migration could also be observed (**Figures 3A,B**).

During the postoperative case management we found that typical neurotrophic ulcers were formed as soon as day 30 after the surgery in all animals in the control (right) limb; moreover, in a number of cases the rats perceived the limb as not belonging to their body that resulted in self-mutilation of digits 3, 4, and 5 of the right extremity, whereas on the contralateral side where ADSCs were transplanted no such complications occurred. Full denervation of limbs to day 60 on both sides resulted in mixed contractures of knee and ankle joints.

Out of 20 animals undergone autoplasty of the sciatic nerve, 4 rats (20%) gnawed off the 3rd, 4th, and 5th fingers of their right paws and sacrificed within a period of up to 1 month. Thus the morphological evaluation on days 30 and 60 involved 16 rats, in two groups of 8 (*n* = 5 – morphological evaluation of regeneration, *n* = 3 – S100, PMP22, and PMP2 mRNA expression).

### Neurons of L5 Spinal Ganglion

**FIGURE 4:**
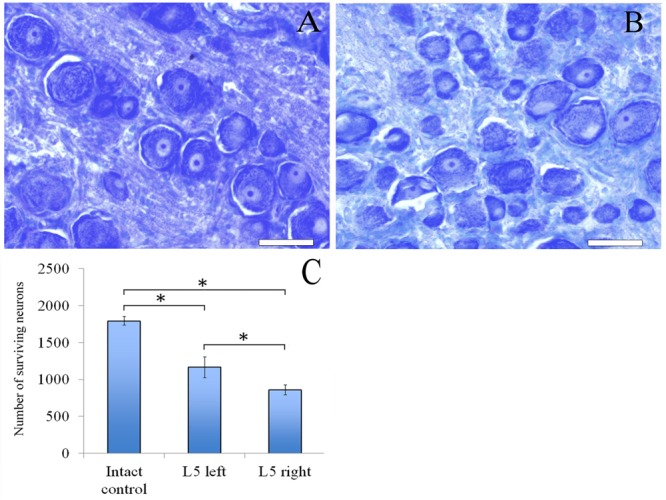
Neurons of L5 spinal ganglion. **(A)** Neurons of L5 left spinal ganglion, **(B)** neurons of L5 right spinal ganglion. Top: histological staining with azure-eosin. Bar: 200 μm. **(C)** Number of surviving neurons of L5 spinal ganglia: 1792.8 ± 58.8 (Intact, *n* = 6), 1166.1 ± 142.3 (L5 left, Experimental, *n* = 5) and 858.3 ± 66.2 (L5 right, Control, *n* = 5). Bottom: quantification of survived neurons of L5 spinal ganglia. Error bars represent standard error mean. Differences were statistically significant between the groups (^∗^), (^∗^*P* < 0.05, one-way ANOVA, Tukey’s test).

**FIGURE 5:**
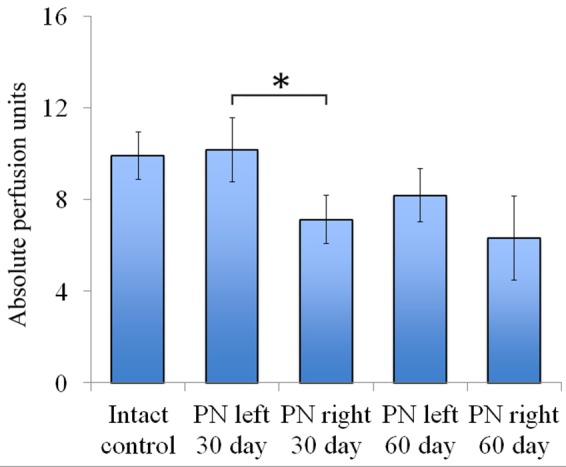
Vascularization 30 and 60 days after the trauma. The distal segment of the sciatic nerve vascularization 30 and 60 days after the trauma. Bottom: apu (absolute perfusion units): 9.92 ± 1.04 (Intact, *n* = 6), 10.18 ± 1.4 (PN left – 30 days, *n* = 8), 7.12 ± 1.06 (PN right – 30 days, *n* = 8), 8.19 ± 1.16 (PN left – 60 days, *n* = 8), 6.32 ± 1.84 (PN right – 60 days, *n* = 8). Error bars represent standard error mean. Differences were statistically significant between the left sciatic nerve (experimental side) and the right sciatic nerve (control side) (^∗^), (^∗^*P* < 0.05, one-way ANOVA, Tukey’s test).

Sixty days after surgery the total number of surviving neurons of L5 spinal ganglia had decreased when compared to the intact animals [1166.1 ± 142.3 (L5 left) and 858.3 ± 66.2 (L5 right) vs. 1792.8 ± 58.8 (Intact)] (**Figure 4C**). The number of surviving neurons of the left spinal ganglia (experimental side) was significantly higher than those in the L5 right spinal ganglia (control side) (**Figure 5**). Despite this difference, there were no significant morphological changes. The right side neurons were more prone to chromatolysis, with cells having an irregular wall structure with cavities between neurons. The cytoplasm of the left spinal ganglia neurons included a granular chromatophilic substance (**Figures 4A,B**).

Assessment of blood flow restoration 30 days after surgery on the left sciatic nerve (experimental side) the parameters of vascularization were higher than in the right sciatic nerve (control side). By day 60 vascularization had slightly decreased when compared to day 30 and never reached the threshold of the intact animals [9.92 ± 1.04 (Intact), 10.18 ± 1.4 (PN left – 30 days), 7.12 ± 1.06 (PN right – 30 days), 8.19 ± 1.16 (PN left – 60 days), 6.32 ± 1.84 (PN right – 60 days)] (**Figure 5**).

### Myelin Fibers

**FIGURE 6:**
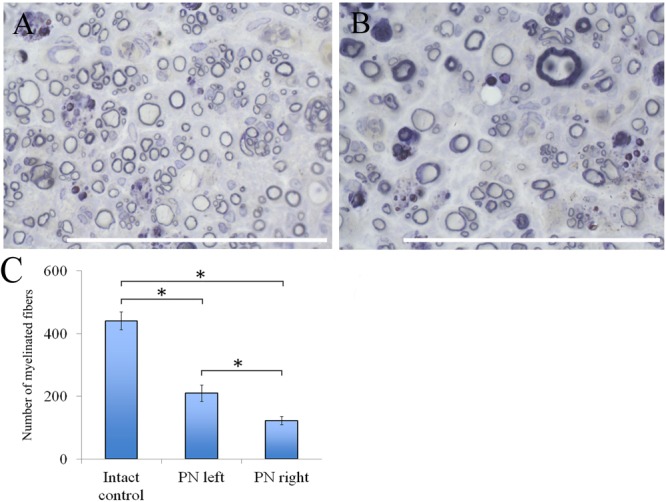
Myelin fibers of the distal segment of the sciatic nerve. **(A)** Myelin fibers of the distal segment of the left sciatic nerve, **(B)** Myelin fibers of the distal segment of the right sciatic nerve. Top: histological staining with osmium and methylene blue. Bar: 50 μm. **(C)** Number of myelinated fibers: 440.67 ± 29.06 (Intact, *n* = 6), 210.17 ± 26.55 (PN left, *n* = 5), 122.17 ± 12.61 (PN right, *n* = 5). Bottom: quantification of myelin fibers of the sciatic nerve. Error bars represent standard error mean. Differences were statistically significant between the groups (^∗^), (^∗^*P* < 0.05, one-way ANOVA, Tukey’s test).

The number of myelin fibers in a distal portion of the nerve after injury significantly decreased when compared to those in the intact animals [440.67 ± 29.06 (Intact) vs. 210.17 ± 26.55 (PN left) vs. 122.17 ± 12.61 (PN right)]. The number of myelin fibers in the left sciatic nerve was 41.87% (*P* < 0.05) higher than in the right one (**Figure 6C**).

Adipose-derived stem cells-induced de- and remyelination processes are clearly visible in the distal part of the left sciatic nerve. There are a lot of Schwann cells forming myelin for one or several nerve fibers. There are degenerating myelin fibers forming large round structures with dark-brown granules. These structures are surrounded by an “onion bulb.” In addition, large blood vessels with a diameter of <50 μm because of a thick endothelium are detected. A sub-endothelial layer, and the middle and outer coats of blood vessels are well-defined. There are numerous formed elements in the lumen of blood vessels.

The portion of the sciatic nerve on the right operated side contains small and medium-sized myelin fibers, round or irregularly shaped, at the remyelination stage as well as non-myelinated fibers. Myelin-forming Schwann cells mainly cover a single myelin fiber. Several Schwann cells with nuclei and myelin fibers are located in fascicle groups separated from each other by interlayers of connective tissue. Between the fascicles of nervous fibers large nuclei of irregularly shaped cells, possibly macrophages, can be seen. Similar cells can be found near or inside the multiple roundish agglomerations consisting of degenerating myelin debris. There also are round structures formed by concentrically flattened myelin fibers and Schwann cells; between these, multiple small diameter capillaries ∼10 μm in diameter were observed. These capillaries are fitted with a single endothelial cell forming one very thin layer. The capillary lumen contains no blood elements (**Figures 6A,B**).

### Transmission Electron Microscopy

**FIGURE 7:**
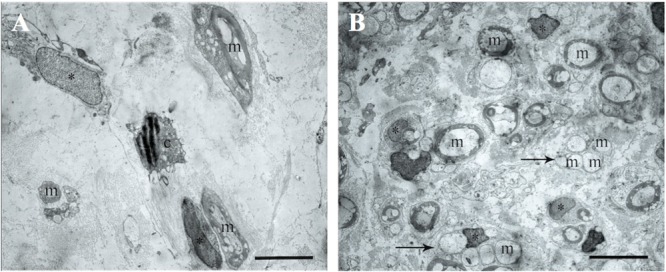
The transmission electron microscopic examination of the distal segment of the right and left sciatic nerve. **(A)** Tissue samples with insert (control group): m- myelin fibers, ^∗^ – nuclei of Schwann cells, light background – connective tissue, c – cells with signs of degeneration, m – myelin fibers. Bar: 5 μm. **(B)** Tissue of the left sciatic nerve samples: m – myelin fibers, ^∗^ – nuclei of Schwann cells, pointer – bundles of myelinated and unmyelinated fibers, n – cell nuclei. Bar: 5 μm.

The electron microscopic examination of the right sciatic nerve revealed that tissue samples with insert (control group) showed significant tissue degeneration. Individual Schwann cells with single myelin fibers and fibrillated structures were found, probably connective tissue in origin (**Figure 7A**).

The transmission electron microscopic examination of the left sciatic nerve samples showed a large amount of myelinated and unmyelinated fibers combined in bundles and forming round structures. No degeneration of these structures was observed. Schwann cells with round nuclei containing euchromatin and cells with elongated or irregularly shaped nuclei containing electron density heterochromatin were also observed. These cells were found between the myelin fibers and were probably fibroblasts (**Figure 7B**).

### S100, PMP22, and PMP2 mRNA Expression in the Spinal Ganglia

**FIGURE 8:**
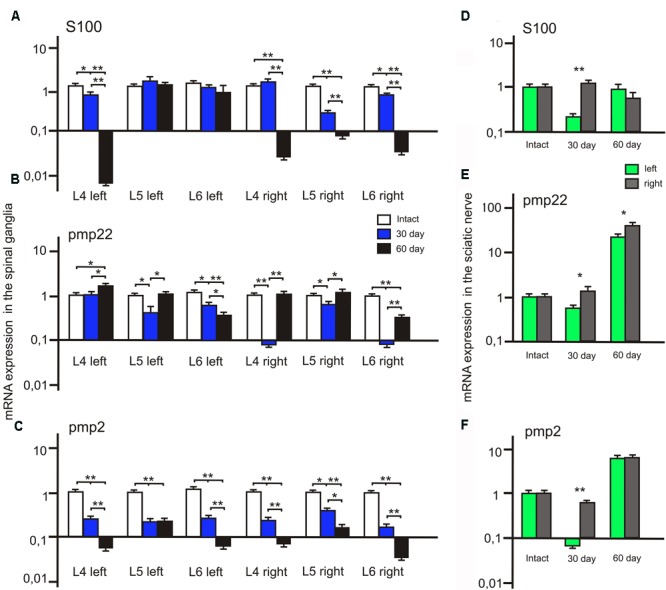
S100, PMP22, and PMP2 mRNA expression in the left (with ADSCs transplantation) and right (without ADSCs transplantation) spinal ganglia **(A–C)** and the sciatic nerves **(D–F)**. Differences were statistically significant between the groups (^∗^). ^∗^*P* < 0.05, ^∗∗^*P* < 0.01, one-way ANOVA, Tukey’s test.

We observed a significant decrease of S100 mRNA expression in the L4 left and L5-6 right spinal ganglia at post-op day 30 (**Figure 8A**). In addition, S100 mRNA expression was >7 times higher in the L5 left compared to the L5 right spinal ganglia. At day 60 S100 mRNA expression decreased in the L4 left and L4-6 right spinal ganglia by 20, 17.5, 0,7 and 14 times compared to the same value at day 30.

After 30 days we observed that the level of PMP22 mRNA expression decreased in the L5-6 left spinal ganglia by 49.2 and 25% (*P* < 0.05) compared to the intact group (**Figure 8C**). However, the difference in PMP22 mRNA expression in the L4, L6 right spinal ganglia at day 30 between the experimental and intact group was more significant [0.09 ± 0.007 (L4 right) vs. 1 ± 0.3 (L4 intact); 0.093 ± 0.009 (L6 right) vs. 1 ± 0.28 (Intact)]. At day 60 PMP22 mRNA expression increased in the L4 left and decreased in the L6 left/right spinal ganglia compared to the intact group (*P* < 0.05).

The level of PMP2 mRNA expression in the L4-6 left and right spinal ganglia as compared to intact animals tends to decrease at all time intervals after the surgery (**Figure 8B**). A significant difference between the left and right spinal ganglia was observed in the L5-6 at day 30 [0.33 ± 0.09 (L5 left) vs. 0.6 ± 0.05 (L5 right); 0.43 ± 0.08 (L6 left) vs. 0.21 ± 0.06 (L6 right)] and in the L6 at day 60 [0.082 ± 0.009 (L6 left) vs. 0.052 ± 0.008 (L6 right)].

### S100, PMP22, and PMP2 mRNA Expression in the Sciatic Nerve

The difference between S100 mRNA expression in the left and right sciatic nerves was only registered at day 30 (**Figure 8D**), whereas in the left portion the expression decreased by 70.3% (*P* < 0.05) as compared to the intact group, in the right part of the graft an expression increase by 32.1% was observed. At day 60 the expression level in the left portion was 13.6% (*P* < 0.05) lower and in the right portion — 32.2% (*P* < 0.05) lower in comparison with the intact nerve. Although S100 mRNA expression in the left sciatic nerves (with ADSCs transplantation) decreased at day 30, we observed the restoration of S100 mRNA expression to the levels of the intact group at day 60. At the same time, in the right sciatic nerves (without ADSCs transplantation) S100 mRNA expression decreased to day 60.

At day 30 PMP22 mRNA expression in the left sciatic nerves (with ADSCs transplantation) decreased by 31.55% (*P* < 0.05) and in the right sciatic nerves (without ADSCs transplantation) increased by 21.19% (*P* < 0.05) in comparison with the intact nerve (**Figure 8F**). At day 60 PMP22 mRNA expression increased manifold both in the left (>33 times) and right (>60 times) sciatic nerves (*P* < 0.01) compared to the intact group.

PMP2 mRNA expression decreases by post -op day 30 by 115% (*P* < 0.05) in the left nerve portion and by 27.9% (*P* < 0.05) in the right (**Figure 8E**). At day 60 there was a significant increase (>7 times) in PMP2 mRNA expression on both operated sides of the peripheral nerve (*P* < 0.05). This fact may be evidence of the initial stage of regeneration as well as myelination, growth, and differentiation.

## Discussion

The proposed model of a nerve injury with its dissected portion transferred to the contralateral side and a critical defect provided serious destructive changes in nerve fibers and blood vessels. Trauma and hypoxia resulted in deleterious changes in the arrangement of axis cylinders that further resulted in neurolysis.

This model is most similar to clinical practice as nerves taken from known regions are used as donors in repairing the integrity of a human nerve trunk. For example, several parts of the sural sensory nerve are used as grafts to repair a defective perineal nerve. However, it does not interfere with the restoration of the lower limb extension function. For this model we use a contralateral nerve as donor and, therefore, its topography does not completely coincide with recipient ends of a reconstructed nerve which allows our relevant conclusions.

The intensity of response of the spinal ganglion sensory neurons correlates to the severity of nerve fiber lesion. It causes slowing down of retrograde axonal transport and neuronal death leading to damage to the working organ feedback path and this is manifested in a decrease in the number of myelin fibers in the peripheral portion of the nerve as compared to intact animals. At the same time, we observed a significant increase of these values on the operated side where allogenic ADSCs were used in every animal. This can be interpreted as proof of their therapeutic potential.

Although we observed a higher grade of regeneration processes in the left sciatic nerve, neuron death as well as the presence of degenerated myelin fibers indicates that this model produces results similar to those seen in clinical practice after a critical defect of the nerve. It is well documented that even under ideal conditions of peripheral nerve posttraumatic regeneration (end-to-end suture and continuity restoration of the nerve trunk in the first hours after an injury) 100% recovery of motor and in particular sensory function cannot be achieved in all cases.

We cannot rule out the transdifferentiation of allogenic ADSCs in Schwann cells that in this case may be a key factor of the higher regeneration effectiveness, but this aspect requires further comprehensive study. We also cannot dismiss the differences in blood flow to reconstructed sciatic nerves, which we have detected with laser doppler. These might be related to activation of the expression of proangiogenic factors by the transplanted cells. This fact has been confirmed by Pelletier et al. (2015) and Lee et al. (2016). We intend to confirm this histologically in further research.

Pathologic signals concerning vessel function cause the formation of ulcers and the animal perceiving its own limb as not belonging to the body and biting off the toes, likely due to the lack of sensibility and/or lack of pain. In case of a sciatic nerve injury on the left and right side trophic ulcers developed only on the operated side not stimulated with ADSCs (Tse et al., 2015). At the present time it is known that the sciatic nerves formed by the processes of motor and sensory neurons also have a trophic function. They have an impact on the intensity of physico-chemical processes in cells and surrounding tissues through the transducers of neuroexcitation — adrenalin, noradrenalin, serotonin, acetylcholine, several amino acids, and neuropeptides getting into efferent cells (Ron et al., 2013). In the case of sciatic nerve transection, the functional stimulation of the innervated structure is terminated due to the disturbance of the neuromediator secretion, and changes occur in secretion or action of comediators and trophogens with trophic effects (Plewnia et al., 1999).

The origin of the trophic factor is still not clear, but it is evident that macromolecular substances of protein, peptide or nucleic nature having a neurotrophic effect and synthesized in neurons, target cells, glial and Schwann cells can be referred to as trophogens (Walsh et al., 2012). They are formed during nerve regeneration, and their activity is regulated through the cell genome. It has been suggested that motor neurons assess the level of trophic support from each of the terminal branches and grow in the direction of the one that provides the greater amount of trophic support (Massing et al., 2010).

These results suggested that all neurons may depend on trophic support derived from their targets for continued survival not only during development but also in the adult nervous system. There is also the possibility that neural precursors and developing neurons whose axons have not contacted their ultimate targets may require trophic support as well (Huang and Reichardt, 2001).

Various nerve growth factors are necessary for the coordinated activity of the nervous system, regulating the state of the synaptic area and acting as receptors and activating the formation of second messengers. Other substances also have an effect on bioelectric and behavioral changes and enhancing neuron regeneration; these can also be classified as trophogens (Mantyh et al., 2011).

It is well known that ADSCs secrete neurotrophic factors (Clauser et al., 2013; Zack-Williams et al., 2015), which probably occurs when they are transplanted into the nerve. According to the literature, proteins of the S100 family are more typical for astrocytes, 10–15% are located in neurons and an significant number in oligodendrocytes (Donato, 2003; Santamaria-Kisiel et al., 2006). In the central nervous system one of the S100 family genes, S100B, facilitates proliferation and inhibits differentiation of astrocytes. An increase in expression is associated with neuronal diseases such as amyotrophic lateral sclerosis and Alzheimer disease (Fujiwara et al., 2014). Depending on their concentration, proteins of the S100 family may have trophic or toxic effects on neurons and glial cells (Woischneck et al., 2010). Our results showed that ADSCs transplantation in the area of suturing of the autologous nerve promotes maintenance of S100 mRNA expression in the spinal ganglia. Although on day 60 this expression in spinal ganglia and the right portion of the nerve is inhibited, we observed an expression increase in the left part of the nerve graft compared to the level of control groups. We believe that expression normalization in the spinal ganglia on day 60 results from the termination of regeneration processes, confirmed by the values of S100 mRNA expression in the sciatic nerve. The absence of overexpression indicates a positive effect of the protein on neurons and glial cells. Based on these results we can also say that in the nerve portion without ADSCs the regeneration induction was recorded already on day 30 but decreased by day 60, whereas in the left portion of the nerve after the introduction of ADSCs the expression level increased significantly at day 60.

Our findings show that gene expression in the spinal ganglia after neuroplasty varies at different periods and in different segments. For example, in the left segment of the sciatic nerve on day 30 the expression of mRNA of PMP22 is decreased as compared to the right segment, likely due to longer lasting axon degeneration after the injury in this area. However, the samples of nerve graft from both sides showed substantial increase of mRNA expression of gene PMP22 already on day 60. We have demonstrated the decrease in PMP2 mRNA expression in the spinal ganglia and the increase in expression in the sciatic nerve. The change in expression level in the sciatic nerve on both operated sides 60 days after the injury confirms lipemic index increase and possible improvement of nerve fiber conduction. In conclusion it seems important to mention that PMP22 protein is the main component of myelin of the peripheral nervous system. Varying expression in the ganglia may be due to the low content of PMP22 protein in the myelin of spinal ganglia, so that it is not representative of PMP22 behavior in the sciatic nerve. This may be due to the myelin sheath disintegration and increased penetration of antibodies, since similar increase of the immune reactivity was found in the nerve fibers undergoing spontaneous Wallerian degeneration in rats (Bolin et al., 1997).

Our results show that the application of ADSCs in the area of suturing of the autologous nerve promotes survival of L5 spinal ganglion neurons and restoration of microcirculation at the site of injury. It also facilitates the growth of myelin fibers throughout the graft area and the maintenance of S100, PMP22, PMP2 mRNA expression in the spinal ganglia.

## Author Contributions

RM and PI: surgery - autologous nerve graft model; cell transplantation; and statistical analysis. GM: morphometric analysis. LM: quantitative analysis of PMP2, PMP22, and S100 gene expression. EG: non-invasive assessment of transplanted cell migration using IVIS Spectrum. SA: electron microscopy. EZ: MSC cultivation. YM: real-time microcirculation visualization using the laser Doppler EasyLDI. ZG: immunofluorescence analysis. VS: MSC transduction by lentivirus coding the eGFP gene. AM: preparation of lentivirus. GK: RNA isolation. MN: confocal microscopy. SM: sample preparation. RY: Epon-Araldite resin embedding. AR: writing the article and analysis of results. ZM: histology sample preparation; extracted the SVF from patient’s fat tissue and counted the number of cells.

## Conflict of Interest Statement

The authors declare that the research was conducted in the absence of any commercial or financial relationships that could be construed as a potential conflict of interest.
